# Parental and Adolescent Educational Expectations and Adolescent Problem Behaviors: The Role of Deviant Peer Affiliations

**DOI:** 10.3390/ijerph20032005

**Published:** 2023-01-21

**Authors:** Yanwen Ouyang, Zirui Ouyang, Xizheng Xu

**Affiliations:** 1 Department of Management, Hunan Police Academy, Changsha 410138, China; 2 School of Educational Science, Hunan Normal University, Changsha 410081, China

**Keywords:** educational expectations, problem behaviors, deviant peer affiliations, parents, adolescents

## Abstract

The comprehensive theory model of problem behaviors proposed that expectations are important factors affecting adolescent problem behaviors. The purpose of this study is to explore the association between educational expectations (in this study, this includes parental educational expectations and adolescent selfeducational expectations) and problem behaviors based on the framework of the CTMPB to provide empirical support for the prevention and intervention of adolescent problem behaviors. This study used cross-sectional data from the 2014–2015 academic year of the China Education Panel Survey (CEPS) conducted by the China survey and data center at the Renmin University of China. A nationwide representative sample of 9936 junior high school students was selected. Among them, 4870 (52.2%) were female, and the average age was 14.52 years (SD = 0.67 years). The results revealed that adolescent selfeducational expectations and deviant peer affiliations played a contributory mediating role in the association between parental educational expectations and adolescent problem behaviors. Both parental educational expectations and adolescent selfeducational expectations are protective factors against adolescent problem behaviors, and enhancing the two factors can decrease the likelihood of adolescent engagement in problem behaviors. In addition, deviant peer affiliations are risk factors for adolescent problem behaviors and represent a mediating factor between educational expectations and adolescent problem behaviors. However, this study was only based on cross-sectional data, requiring further support by longitudinal or experimental studies.

## 1. Introduction

Problem behaviors are conceptualized as “abnormal” behaviors that hinder individuals’ social adaptation [1], violate social norms, fail to adapt to social life and make a negative impact on or harm communities and society [2]. These problem behaviors include aggressive behaviors, delinquent behaviors, rule-breaking behaviors, and so on. Adolescent problem behaviors are major mental and physical health issues in adolescents and have been an important research topic for a long time.

The comprehensive theoretical model of problem behaviors (CTMPB) pointed out that expectations are important factors affecting adolescent problem behaviors [3]. Educational expectations, included in the “expectations system”, generally refer to the hopes and expectations for achieving the highest level of education [4]. For adolescents, educational expectations mainly include adolescent selfeducational expectations and parental educational expectations. Previous research on educational expectations mainly focused on the association between educational expectations and academic performance. A large number of studies found that educational expectations can significantly predict academic performance and future education levels [5,6,7,8]. However, the role of educational expectations seems to go far beyond that. Some recent studies also found that educational expectations might be related to problem behaviors [9,10], but rare empirical studies focused on the association between educational expectations and adolescent problem behaviors. In order to fill this gap, this study aims to further affirm and explore the association between educational expectations and adolescent problem behaviors based on the framework of the CTMPB.

## 2. Theoretical Background

### 2.1. The Comprehensive Theoretical Model of Problem Behaviors

Jessor et al. established the CTMPB based on the two-factor theory and the problem–behavior theory [9]. The CTMPB is based on a theoretically derived conceptualization that incorporates both contextual and individual differences in protection and risk; the model takes into account both the direct effects of protective and risk factors and the moderating influence that protection may have on the impact of exposure to risk [3]. The CTMPB reflects the thoughts of the ecological systems theory and positive psychology, which give a better account of the problem behavior and go a step ahead of the two-factor theory and the problem-behavior theory.

**Figure 1:**
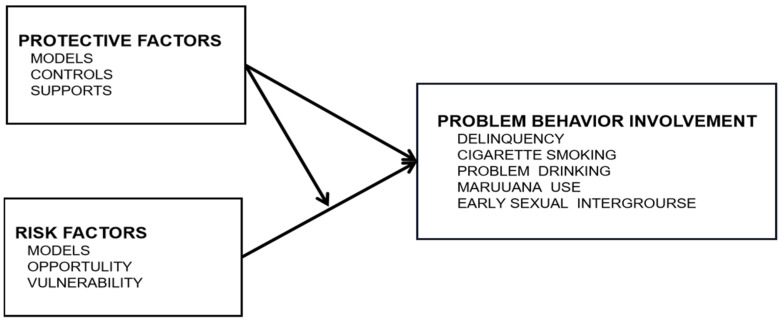
The comprehensive theoretical model of problem behaviors.

The CTMPB was developed as a risk-protection model, which includes three types of protection (models protection, controls protection, and support protection) and three types of risk (models, opportunity, and vulnerability risks), including not only the measures of individual-level protection and risk but also measures of protection and risk in the multiple social contexts that are salient in the ecology of daily adolescent life: family, peers, school, and the neighborhood. Conceptually, protective factors decrease the likelihood of engaging in problem behaviors; risk factors, in contrast, increase the likelihood of engaging in problem behavior by providing models for problem behavior, greater opportunity for engaging in problem behavior, and greater personal vulnerability to problem behavior involvement. In addition, protective factors can play an additional—indirect—role in the occurrence of adolescent problem behavior by moderating or buffering the impact of risk factors [3]. The interaction of factors in CTMPB can be seen in Figure 1.

### 2.2. Parental Educational Expectations and Adolescent Problem Behaviors

According to CTMPB, although the “parental educational expectations” have not been pointed out as factors impacting adolescent problem behaviors, there are three types of protection (models protection, controls protection, and support protection) [3]. In order to help adolescents realize their educational goals, parents must be motivated by their educational expectations to invest more resources, such as providing better conditions for adolescent education, guiding and monitoring adolescent behaviors, and so on. Therefore, these kinds of parent behaviors that relate to educational expectations, in fact, might become the above “protection” against adolescent problem behaviors.

One of our recent studies found that parental educational expectations are significantly correlated with adolescent problem behaviors, and parental educational expectations can significantly negatively predict adolescent problem behaviors. The study, based on a nationwide sample of 6888 participants: 3342 (48.5%) were female, and 3546 (51.5%) were male with an average age of 14.50 years (SD = 0.68 years), aimed to explore the mechanism by which family socioeconomic status influences adolescent problem behaviors. The results revealed that parental educational expectations and adolescent confidence in the future played a contributory mediating role in the association between family socioeconomic status and adolescent problem behaviors (standardized path coefficient = 0.124, *p* < 0.001). High parental educational expectations are protective factors against adolescent problem behaviors [9].

In addition, there exists an earlier study that supported the above conclusion. A study conducted by John Mark Froiland and Mark L. Davison (the participants were 6th- to 12th-grade students from 5828 families across the US), aiming to explore the influence of parental expectations and school relationships on adolescent outcomes, revealed that parental expectations were positively related (standardized path coefficient = 0.44, *p* < 0.01) to positive school outcomes. In this study, problem behaviors at school are one of three dimensions of the school outcome variables; the other two dimensions are students’ grades and retention in any grade [10]. According to the CTMPB and the above experiment studies, we propose the hypothesis as follows.

**Hypothesis 1 (H1).**  *Parental educational expectations significantly negatively predict adolescent problem behaviors.*

### 2.3. The Mediating Role of Adolescent Selfeducational Expectations

According to the expectation network theory, the expectations of others (including parental expectations) can directly or indirectly affect the behaviors of individuals through their selfexpectations [11]. On the mechanism by which parents’ educational expectations influence their children’s educational expectations, some scholars found that parental expectations have a self-fulfilling prophecy effect on their children’s educational performance [12], namely when adolescents are exposed to external stimuli, they might define and treat it as if it were true.

There are many previous studies that have illustrated that there is a significant correlation between the parental educational expectations level and the adolescent educational expectations level. The findings of these studies showed that parental educational expectations are important factors influencing adolescent selfeducational expectations; parental educational expectations can significantly predict adolescent selfeducational expectations [13,14,15]. For instance, a study conducted by Kirk, CM et al. revealed that educational expectations in adolescents were indeed predicted by their parents’ educational expectations (F (1, 170) = 33.22, *p* < 0.001, r^2^ = 0.164, adjusted r^2^ = 0.159) [14]. At the same time, there is also evidence that shows that even though educational expectations experience a process of intergenerational interaction and transmission in the family environment, there is a general difference between adolescent educational expectations and parental educational expectations [16,17].

According to the CTMPB, there are two kinds of factors affecting problem behaviors: one is the situational system factors, which are known as the distal variables and have an indirect impact on problem behaviors. The other is the personality system factors, which are known as proximal variables and have a more direct impact on problem behaviors. The variables constituting the personality system are at the level of social cognition and include values, expectations, beliefs, attitudes, and orientations of self and others [3]. “Expectations” were suggested as important factors impacting adolescent problem behaviors in the personality system in the CTMPB. From the CTMPB and the expectation network theory and the above experiment studies, we propose the hypothesis as follows.

**Hypothesis 2 (H2).**  *Adolescent selfeducational expectations play a mediating role between parental educational expectations and adolescent problem behaviors.*

### 2.4. The Mediating Role of Deviant Peer Affiliations

According to the CTMPB, risk factors increase the likelihood of engagement in problem behaviors, and there are also three types of risks (models, opportunity, and vulnerability risk) [3]; model risks include measures such as peer models for alcohol use; opportunity risks include opportunity measures such as the availability of alcohol in the home; vulnerability risks include personal vulnerability measures such as feelings of peer stress. These risks might exist in peer interactions. It is widely acknowledged that peers play an integral role in adolescent behaviors [18]. As early adolescents begin to rely more on peers for critical sources of identity, selfevaluation, and personal worth, they become more responsive to peer influence [19]. A large body of research has examined the role of negative peer influence, such as associations with peers who engage in delinquent or aggressive behavior, on adolescent problem behaviors [20,21,22,23,24,25]. For instance, a study conducted by Zhu J et al. revealed that deviant peer affiliation was positively correlated with problem behaviors (r = 0.42, *p* < 0.01) [25].

Meanwhile, some studies have found that adolescent selfeducational expectations may be important factors influencing the peer affiliations of adolescents. According to status socialization theory [26], adolescent selfeducational expectations build the mental bases for everyday commitments that guide behavior in and outside of school towards higher-order educational goals [27], thus enhancing adolescent selfefficacy [28] and selfcontrol and are more likely to avoid deviant peer affiliations. The theory of secondary homophily holds that [29] adolescents tend to befriend peers with the same ethnic and racial backgrounds [30], similar school grades [31,32], and similar socioeconomic status [33] and these attributes potentially intersect with educational expectations, and might increase the expectation-related sorting of students within friendship networks [27]. Studies demonstrated that adolescents tend to select friends with the same educational expectations [27,34]. Furthermore, a recent study result showed that the selection effect is more significant than the influence effect in the relationship between educational expectations and adolescent friendships [35]. According to the above experiment studies and the CTMPB and status socialization theory, we propose the hypothesis as follows:

**Hypothesis 3 (H3).**  *Deviant peer affiliations play a mediating role between adolescent selfeducational expectations and adolescent problem behaviors.*

As mentioned above, to help adolescents realize their educational goals, parents must be motivated by their educational expectations to invest in more resources. Parents will not only invest more economic resources but also enhance the monitoring of their children. Some studies have found that parental monitoring is an important factor affecting adolescents deviant peer affiliations [36,37]; a study conducted by Lin S. revealed that parental monitoring observed in the 7th grade predicted deviant peer affiliation in the 8th grade (β = 0.23, *p* < 0.01) [36]; coupled with the intergenerational transmission between parental educational expectations and adolescent selfeducational expectations, we make a further hypothesis as follows:

**Hypothesis 4 (H4).**  *Deviant peer affiliations play a mediating role between parental educational expectations and adolescent problem behaviors.*

## 3. Materials and Methods

The purpose of this study is to affirm the association between educational expectations and adolescent problem behaviors and explore the more protective or risk-creating factors for adolescent problem behaviors based on the framework of the CTMPB and to provide empirical supports for the prevention and intervention of adolescent problem behaviors.

### 3.1. Participants

This study used cross-sectional data from the 2014–2015 academic year of the China Education Panel Survey (CEPS) conducted by the China survey and data center at the Renmin University of China. The survey uses a multistage probability-to-scale (PPS) sampling method. At the first sampling stage, 28 counties (districts) were selected from the national county (district) level administrative units. At the second sampling stage, four schools with grade 7 and/or grade 9 were selected from the geographical area of each enrolled county (district). At the third sampling stage, four classes were selected from each school, including two grade 7 classes and two grade 9 classes. A total of 438 classes in 112 schools were randomly selected from the selected county-level units. At the fourth sampling stage, all students, parents, head teachers, teachers of a main subject (i.e., English and Chinese), and school leaders of the enrolled class constitute the final survey samples. The study was approved by the Ethics Committee of the Renmin University of China, and all participants were informed that their participation would be voluntary and that they were free to withdraw from the study at any time. The anonymity of the study was also emphasized before the data were collected. Participants were asked to respond independently according to their actual empirical situation. Due to the missing data, a total of 9936 students in the second grade of junior high school were finally included in this study. Among them, 4870 (52.2%) were female, and 4466 (47.8%) were male, and their average age was 14.52 years (SD = 0.67 years).

### 3.2. Measures

#### 3.2.1. Parental Educational Expectations

Parental educational expectations were measured by one question: “Which level of education do your parents want you to achieve in the future?” and the corresponding answers of “give up school now” to “PhD”. After referring to another study on educational expectations [38], this study took into consideration the “indifferent” option corresponding to the “give up school now” option in the questionnaire as the lowest expectation. Technical secondary schools, technical schools, vocational high schools, and general high schools are classified as part of the same grade because they have the same number of education years. Finally, there was a 7-point Likert scale, wherein higher scores indicate a higher level of education.

#### 3.2.2. Adolescent Problem Behaviors

Adolescent problem behaviors were measured by the problem behaviors questionnaire from the 2015 student questionnaire of the CEPS program. The questionnaire consisted of 7 questions (After the confirmatory factor analysis, the original 10 questions were reduced to 7 questions), such as “Have you done the following behaviors in the past year? “To fight” was represented on a 5-point Likert scale, where 1 = “never”; 2 = “occasionally”; 3 = “sometimes”; 4 = “often”; 5 = “always”. The Cronbach’s coefficient of the whole scale is 0.75. The results of scale confirmatory factor analysis showed that CFI = 0.943 > 0.9, TLI = 0.915 > 0.9, RMSEA = 0.082 < 0.1, and SRMR = 0.038 < 0.05.

#### 3.2.3. Adolescent Self-Educational Expectations

Adolescent educational expectations were measured by one question, “Which level of education do you want to achieve in the future?” and the corresponding answers were “give up school now” to “ PhD”. After referring to another study on educational expectations [38], this study took into consideration the “indifferent” option corresponding to the “give up school now” option in the questionnaire as the lowest expectation. Technical secondary schools, technical schools, vocational high schools, and general high schools are classified as part of the same grade because they have the same number of education years. Finally, there was a 7-point Likert scale, wherein higher scores indicate a higher level of education.

#### 3.2.4. Deviant Peer Affiliations

Deviant peer affiliations were measured by the deviant peer affiliations questionnaire from the 2015 student questionnaire of the CEPS program. The questionnaire consists of 7 questions, such as “How many friends of yours have done the following behaviors? “Smoking and drinking” was represented on a 3-point scale, where 1 = “no one”; 2 = “one or two”; 3 = “many”. The Cronbach’s coefficient of the whole scale is 0.88. The results of scale confirmatory factor analysis showed that CFI = 0.980 > 0.9, TLI = 0.969 > 0.9, RMSEA = 0.071 < 0.08, and SRMR = 0.020 < 0.05.

## 4. Result Analysis and Discussion

### 4.1. Result Analysis

This study used SPSS 26.0 (International Business Machines Corp, US) and PROCESS V3.5 (Linda Muthén & Bengt Muthén, US) and Mplus VERSION 8.3. Model 6 in PROCESS V3.5 was selected for testing. Mplus VERSION 8.3 was used for the scale confirmatory factor analysis. Our analyses were presented in three parts, which include, descriptive statistics and correlations including the means, standard deviations, and Pearson correlation coefficients of the main variables. The regression models analysis included four equations and regression coefficients, standard errors, and *p* values; Finally, the mediating role analysis includes the ratio of three indirect paths and their comparisons.

#### 4.1.1. Descriptive Statistics and Correlations

Table 1 presents the descriptive statistics and correlation analysis results of each variable. It shows that there were significant correlations among all the variables, and parental educational expectations and adolescent selfeducational expectations are negatively correlated with deviant peer affiliations and adolescent problem behaviors. In addition, the correlation coefficient between parental educational expectation and adolescent selfeducation expectation was the highest (0.658, *p* < 0.001), followed by the correlation coefficient of deviant peer affiliations and adolescent problem behavior (0.479, *p* < 0.001).

Previous studies have found that gender and parental relationships have an impact on adolescent problem behaviors [39,40,41], so gender and parental relationships were controlled in this study as covariates and were included in the correlation analysis.

#### 4.1.2. Regression Models Analysis

The results of the correlation analysis meet the statistical requirements for further testing the mediating role of adolescent selfeducational expectations and deviant peer affiliations [42]. Furthermore, the SPSS macro program developed by Hayes [43] was used to perform the bootstrap-based mediating role test using model 6.

Table 2 shows an analysis of the results of the regression relationship among the variables. First, Equation 1 indicated that parental educational expectations significantly negatively predicted adolescent problem behaviors (*β* = −0.194, *p* < 0.001), and when adolescent selfeducational expectations and deviant peer affiliations were included in the regression equation, Equation 4 showed that adolescent selfeducational expectations also negatively predicted adolescent problem behaviors (β = −0.155, *p* < 0.001). However, at this time, the significance level of parental educational expectations predicting adolescent problem behaviors lowered (β = −0.033, *p* = 0.005). Second, Equation 3 indicated that parental educational expectations negatively predicted deviant peer affiliations (β = −0.060, *p* < 0.001), and adolescent selfeducational expectations also negatively predicted deviant peer affiliations (β = −0.130, *p* < 0.001). Third, Equation 2 showed that there were high regression coefficients between parental educational expectations and adolescent selfeducational expectations and between deviant peer affiliations and adolescent problem behaviors.

#### 4.1.3. Mediating Role Analysis

**Figure 2:**
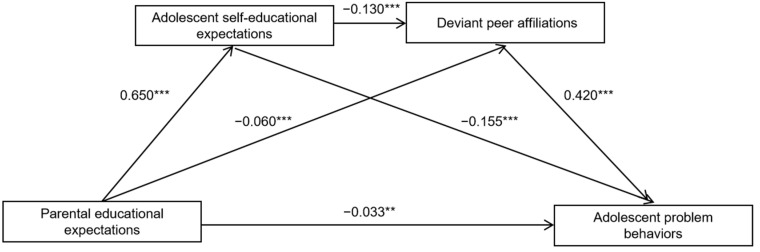
The paths of the multiple mediations. ** *p* < 0.01, *** *p* < 0.001.

Table 3 and Figure 2 show that adolescent educational expectations and deviant peer affiliations played a significant mediating role in the link between parental educational expectations and adolescent problem behaviors. The total standardized mediating regression coefficient was −0.161. The mediating role was composed of the following three indirect paths: Indirect path 1: the path of parental educational expectations, adolescent selfeducational expectations, and adolescent problem behaviors; Indirect path 2: the path of parental educational expectations, deviant peer affiliations, and adolescent problem behaviors, and Indirect path 3: the path of parental educational expectations, adolescent educational expectations, deviant peer affiliations, and adolescent problem behaviors. The ratios of the three indirect paths to the total are 62.73%, 15.53%, and 21.74%, respectively. All three indirect paths are significant.

The indirect paths comparison option in Model 6 was selected, and the indirect paths of the different pathways were compared pairwise to investigate whether there were significant path differences between the pathways. Comparison 1 showed that the bootstrap 95% confidence interval for the difference between indirect paths 1 and 2 did not include a 0 value, indicating that there was significant difference between indirect paths 1 and 2. Comparison 2 showed thatthe bootstrap 95% confidence intervals for the differences between indirect paths 1 and 3 did not include a 0 value, indicating that there was significant difference between indirect paths 1 and path3 too. But Comparison 3 showed the bootstrap 95% confidence intervals for the differences between indirect paths 2 and 3 included a value of 0, indicating there is no significant difference between paths 2 and 3.

### 4.2. Discussion

This study explored the association between educational expectations and adolescent problem behaviors based on a nationwide representative sample from China. We examined the association between parental educational expectations and adolescent problem behaviors and analyzed the mediating roles of adolescent selfeducational expectations and deviant peer affiliations.

#### 4.2.1. The Association between Parental Educational Expectations and Adolescent Problem Behaviors

The findings of this study revealed that parental educational expectations can significantly predict adolescent problem behaviors. “Equation 1” in Table 2 indicated the association between parental educational expectations and adolescent problem behaviors (*β* = −0.161, *p* < 0.001), namely parental educational expectations that significantly negatively predict adolescent problem behaviors, which supports H1. This result is consistent with previous research results [9,10], and the result of this study also extended the range of the CTMPB risk-protection model variables; parental educational expectations, as situational system factors, can be “protective factors” against adolescent problem behaviors [3].

These findings revealed the importance of parental educational expectations on adolescents, not only in the context of adolescent academic achievement and future educational attainment, as the past studies suggested, but also in the context of adolescent development. High parental educational expectations might enhance “support protection” (for example, accompanying children) and “control protection” (for example, parent monitoring) against adolescent problem behaviors [3]. Therefore, enhancing parental educational expectations means reducing the likelihood of engagement in problem behaviors. Conversely, if parents have lower educational expectations for their children for whatever reasons, the consequence might not only decrease adolescent academic performance and future educational attainment but also increase the adolescents’ risk of expressing problem behaviors.

#### 4.2.2. The Parallel Mediating Role of Adolescent Selfeducational Expectations and Deviant Peer Affiliations

The results of this study revealed that adolescent selfeducational expectations and deviant peer affiliations play a parallel mediating role in the link between parental educational expectations and adolescent problem behaviors. Indirect path 1 from Table 3 (parental educational expectations, selfeducational expectations, and adolescent problem behaviors; *β* = −0.101, Boot LLCI = −0.120, Boot ULC = −0.081) and Indirect path 2 (parental educational expectations, deviant peer affiliations, and adolescent problem behaviors; *β* = −0.025, Boot LLCI = −0.039, Boot ULCI = −0.011) indicated the mediating role of adolescent selfeducational expectations and deviant peer affiliations played on the link between parental educational expectations and adolescent problem behaviors, which supports H2 and H4.

Equation 2 (*β* = 0.650, *p* < 0.001) indicated the association between parental educational expectations and adolescent selfeducational expectations. These results support the expectation network theory, which holds that parental expectations can directly or indirectly affect behavior through adolescent selfexpectations [11]. Equation 3 (*β* = −0.060, *p* < 0.001) indicated the association between parental educational expectations and deviant peer affiliations, and parental educational expectations can directly significantly predict deviant peer affiliations.

Equation 4 (*β* = −0.130, *p* < 0.001) indicated the association between selfeducational expectations and adolescent problem behaviors, showing that adolescent selfeducational expectations can significantly negatively predict adolescent problem behaviors. This result supports the CTMPB, which argued that adolescent selfeducational expectations, as personality system factors, influence adolescent problem behaviors. This result further suggests that the high selfeducational expectations of adolescents are protective factors against adolescent problem behaviors. Equation 4 (*β* = 0.420, *p* < 0.001) indicated the association between deviant peer affiliations and adolescent problem behaviors, and deviant peer affiliations can significantly negatively predict adolescent problem behaviors. This result is consistent with previous research results [18,19,20,21,22,23,24], wherein deviant peer affiliations represent a crucial risk factor for adolescent problem behaviors.

#### 4.2.3. The Chain Mediating Role of Adolescent Selfeducational Expectations and Deviant Peer Affiliations

The findings of this study revealed that, apart from the two above paths, there is a third path. Indirect path 3 from Table 3 (parental educational expectations, adolescent selfeducational expectations, deviant peer affiliations, and adolescent problem behaviors; *β* = −0.035, Boot LLCI = −0.046, Boot ULCI = −0.025) indicated the chain mediating role of adolescent selfeducational expectations and deviant peer affiliations on the link between parental educational expectations and adolescent problem behaviors, which supports H3.

Equation 3 indicated that adolescent selfeducational expectations can significantly negatively predict deviant peer affiliations. This result is consistent with previous studies [27,34] and revealed that adolescents tend to choose peers who are at the same level of educational expectations to make friends, and thus adolescents with high selfeducational expectations are more likely to focus on academic achievement, thus are more likely to avoid deviant peer affiliations. The result shows that high selfeducational expectations, as protective factors again adolescent problem behaviors, can decrease the likelihood of engagement in problem behaviors by decreasing deviant peer affiliations.

Finally, it is worth mentioning that Equation 4 indicated a direct path coefficient (*β* = −0.033, *p* = 0.005) for the association between parental education expectations; this result shows that adolescent selfeducational expectations and deviant peer affiliations play most of the mediating role in the link between parental educational expectations and adolescent problem behaviors based on the condition that the covariates are parental relationship and gender.

## 5. Implications and Limitations of the Research

The findings of this study have some implications for adolescent problem behavior prevention and intervention. Both parental educational expectations and adolescent selfeducational expectations are conducive to adolescent development. Parents should pay more attention to their educational expectations of their adolescent children and enhance adolescent selfeducational expectations through various ways to avoid deviant peer affiliations and reduce the likelihood of adolescent engagement in problem behaviors. The parents of adolescents with problem behaviors, in particular, should know that lower educational expectations might increase the risk of adolescent engagement in problem behaviors.

This study also has some limitations. This study is only based on cross-sectional data, and it is difficult to determine the causal relationship between the variables; future research could be enriched by dynamic analyses. Moreover, according to the CTMPB, there are three types of protection and three types of risks [10]. Future research could extend to various types of protection and take more factors into account, such as parental controls and good peer models. In addition, more of the protective and risk factors between adolescent selfeducational expectations and problem behaviors need to be further explored.

## 6. Conclusions

Parents’ educational expectations and adolescents’ selfeducational expectations are important factors related to adolescent problem behaviors. Both parental educational expectations and adolescent selfeducational expectations can significantly negatively predict adolescent problem behaviors. Both high parental educational expectations and adolescent selfeducational expectations are protective factors against adolescent problem behaviors. Adolescent deviant affiliations are risk factors and play mediating roles in the link between parental educational expectations and adolescent problem behaviors, as well as the link between adolescent selfeducational expectations and adolescent problem behaviors.

There might be a way to prevent and intervene in adolescent problem behaviors, that is, to enhance parental educational expectations and adolescent selfeducational expectations to avoid deviant peer affiliations and reduce the likelihood of engagement in problem behaviors. But this study is only based on cross-sectional data; this point needs to be further supported by longitudinal or experimental studies.

## Figures and Tables

**Table 1:** Means, standard deviations, and Pearson correlation coefficients of main variables.

	M	SD	1	2	3	4	5
1 Parental educational expectation	4.67	1.43					
2 Adolescent educational expectations	4.73	1.50	0.658 ***				
3 Deviant peer affiliations	8.23	2.33	−0.168 ***	−0.199 ***			
4 Adolescent problem behaviors	15.54	4.84	−0.218 ***	−0.276 ***	0.479 ***		
5 Parental relationships	0.90	0.30	0.068 ***	0.074 ***	−0.068 ***	−0.107 ***	

Note: N = 9936; *** *p* < 0.001. Gender was dummy coded so that 0 represented the females and 1 represents the males.

**Table 2:** Regression equations.

Variables	Equation 1		Equation 2		Equation 3		Equation 4	
	*β*	*t*	*β*	*t*	*β*	*t*	*β*	*t*
Parental educational expectations	−0.194 ***	−19.471	0.650 ***	83.174	−0.060 ***	−4.533	−0.033 *	−2.797
Adolescent educational expectations					−0.130 ***	−9.790	−0.155 ***	−13.090
Deviant peer affiliations							0.420 ***	45.590
Parental relationships	−0.99 ***	−9.965	0.032 ***	4.057	−0.059 ***	−5.947	−0.067 ***	−7.593
Gender	−0.180 ***	−18.101	0.067 ***	8.595	−0.196 ***	−19.560	−0.084 ***	−9.215
*R*	0.297		0.663		0.287		0.524	
*R* ^2^	0.088		0.439		0.082		0.274	
*F*	301.163 ***		2433.368 ***		209.324 ***		705.921 ***	

Note: Equation 1 = adolescent problem behaviors as the dependent variable; Equation 2 = adolescent educational expectation as the dependent variable; Equation 3 = deviant peer affiliations as the dependent variable; Equation 4 = adolescent problem behaviors as the dependent variable. All variables in the model have been standardized. * *p* < 0.01, *** *p* < 0.001.

**Table 3:** Mediating paths comparison.

	Regression Coefficient	Boot SE	Boot LLCI	Boot ULCI	Percentage
Total indirect path	−0.161	0.011	−0.182	−0.140	100%
Indirect path 1	−0.101	0.010	−0.120	−0.081	62.73%
Indirect path 2	−0.025	0.007	−0.039	−0.011	15.53%
Indirect path 3	−0.035	0.005	−0.046	−0.025	21.74%
Comparison 1	−0.076	0.012	−0.100	−0.052	
Comparison 2	−0.065	0.011	−0.087	−0.043	
Comparison 3	0.010	0.011	−0.011	0.033	

Note: Boot SE, Boot LLCI, and Boot ULCI refer to standard errors and lower and upper limits of the 95% confidence intervals for the indirect paths estimated by the bias-corrected percentile bootstrap method (5000 times), respectively. Indirect path 1: parental educational expectations, adolescent selfeducational expectations, and adolescent’ problem behaviors; Indirect path 2: parental educational expectations, deviant peer affiliations, and adolescent problem behaviors; Indirect path 3: parental educational expectations, adolescent selfeducational expectations, deviant peer affiliations, and adolescent problem behaviors.

## Data Availability

The data presented in this study are available on request from the corresponding author. The data are not publicly available due to privacy.
